# Beating the Fault-Tolerance Bound and Security Loopholes for Byzantine Agreement with a Quantum Solution

**DOI:** 10.34133/research.0272

**Published:** 2023-11-21

**Authors:** Chen-Xun Weng, Rui-Qi Gao, Yu Bao, Bing-Hong Li, Wen-Bo Liu, Yuan-Mei Xie, Yu-Shuo Lu, Hua-Lei Yin, Zeng-Bing Chen

**Affiliations:** ^1^ National Laboratory of Solid State Microstructures and School of Physics, Collaborative Innovation Center of Advanced Microstructures, Nanjing University, Nanjing 210093, China.; ^2^ Department of Physics and Beijing Key Laboratory of Opto-electronic Functional Materials and Micro-nano Devices, Key Laboratory of Quantum State Construction and Manipulation (Ministry of Education), Renmin University of China, Beijing 100872, China.

## Abstract

Byzantine agreement, the underlying core of blockchain, aims to make every node in a decentralized network reach consensus. Classical Byzantine agreements unavoidably face two major problems. One is 1/3 fault-tolerance bound, which means that the system to tolerate *f* malicious players requires at least 3*f* + 1 players. The other is the security loopholes from its classical cryptography methods. Here, we propose a Byzantine agreement framework with unconditional security to break this bound with nearly 1/2 fault tolerance due to multiparty correlation provided by quantum digital signatures. It is intriguing that quantum entanglement is not necessary to break the 1/3 fault-tolerance bound, and we show that weaker correlation, such as asymmetric relationship of quantum digital signature, can also work. Our work strictly obeys two Byzantine conditions and can be extended to any number of players without requirements for multiparticle entanglement. We experimentally demonstrate three-party and five-party consensus for a digital ledger. Our work indicates the quantum advantage in terms of consensus problems and suggests an important avenue for quantum blockchain and quantum consensus networks.

## Introduction

Byzantine agreement requires solving the fundamental consensus problem initially posed in 1982 known as the Byzantine Generals Problem, which can ensure the smooth functioning of a decentralized system under the attacks of malicious players [1,2]. This problem can be translated into a “commanding general-lieutenants” model, where the commanding general is randomly selected from among all the Byzantine generals and the others become lieutenants to reach consensus on the commanding's order (see Supplementary Materials Section A for details). For a strict Byzantine agreement, there are two necessary interactive consistency (IC) Byzantine conditions. The first is that all loyal lieutenants obey the same order (IC_1_), and the second is that every loyal lieutenant obeys the order of the commanding general if the commanding general is loyal (IC_2_). Only when both conditions are satisfied can the system reach consensus. For an *N*-party system, however, classical Byzantine agreement (CBA) protocols [3–9] that tolerate *f* malicious players require *N* ≥ 3*f* + 1 players; namely, the fault-tolerance bound is 1/3 [10–13]. Thus, the three-party consensus problem is naturally unsolvable for CBA even using the authentication classical channel [14]. The other issue is the security loopholes of CBA’s widely used public-key encryption and one-way hash function [15], which are seriously threatened by quantum computing [16–25].

Quantum Byzantine agreement (QBA) is a promising approach for consensus problems. For three-party consensus, the first quantum solution using three-qutrit singlet states was proposed in 2001 [26] and was experimentally demonstrated using four-photon polarization-entangled states in 2008 [27]. This protocol and its subsequent protocols [28–32] using some special entanglement, called detectable QBA framework, unavoidably weaken the two original Byzantine conditions with extra assumptions, which leads to a certain probability of aborting the protocol. More seriously, these rudimentary solutions are restricted to the three-party scenario and can only reach a one-bit message consensus [26,27,29–32]. Some achievements have been made toward scalable multiparty QBA [33–36], but their fault tolerance is 1/3. In addition, QBA protocols require sophisticated techniques, such as multiparticle entanglement generation and distribution and entanglement swapping, which are difficult for practical implementations. Furthermore, the security of detectable QBA has not been proven rigorously [37,38].

Intriguingly, quantum entanglement is not necessary to break the 1/3 fault-tolerance bound and weaker correlation can also work. Here, different from detectable QBA, we propose a strict information-theoretical secure Byzantine agreement framework that exploits the recursion structure [39] and quantum digital signatures (QDS) [40–42] to address the limitation of fault-tolerance bound and security loopholes (see Table 1). It completely breaks the 1/3 fault-tolerance bound with a fault tolerance of *N* ≥ 2*f* + 1, ∀ *f* ∈ *ℕ*^+^ while strictly obeying IC_1_ and IC_2_ due to multiparty correlation provided by QDS. Our work is highly adaptable, because it can be achieved by any type of QDS, including the original proposal of GC01-QDS [40] and its variants such as orthogonal encoding [43–51] and non-orthogonal encoding [52–55], and OTUH-type QDS [56,57]. As QDS advances by leaps and bounds, it only requires coherent states instead of complex multiparticle entanglement and quantum memory [41,43,52]. Generating and maintaining entanglement is a sticking point in experimental setups, and the ability to relax this requirement can reduce the complexity of consensus systems and serve as a foundation for further research. Furthermore, our protocol is able to achieve consensus on multiple messages. In addition, we implement proof-of-principle experiments of the three-party and five-party consensus with three different QDS protocols, BB84 GC01-QDS [43], OTUH-QDS [56], and OTUH-QDS without perfect keys [57].

**Table 1.** Comparison between our work and detectable QBA framework. D-QBA, detectable QBA; N/A, not applicable; IC_1_ and IC_2_, two interactive consistency Byzantine conditions

Performance	This work	D-QBA [26–32]
Security analysis	Yes	N/A
Fault tolerance	*N* ≥ 2*f* + 1, ∀ *f* ∈ ℕ^+^	*N* = 3, *f* = 1
Message	Multiple	Binary
Decentralization	Yes	N/A
Entanglement	No	Yes
Strictly obey IC_1_ and IC_2_	Yes	No

## Results

### Protocol definition

**Fig. 1.**
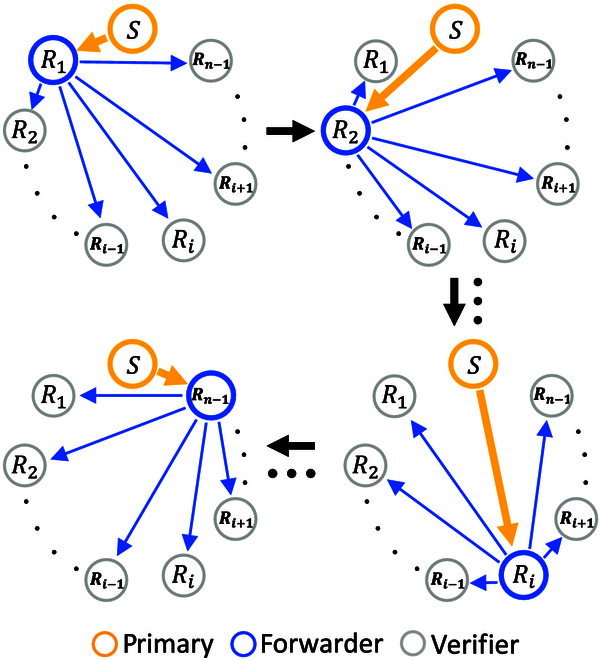
Schematic of a multicast round including *n* players with primary *S*. The primary *S* signs and then multicasts his message to the backups. The forwarder is chosen from among the backups, and unchosen backups act as verifiers. The primary, the forwarder, and one of the verifiers perform a three-party QDS. The backups take turns acting as the forwarder. The arrow indicates the direction of the message delivery.

Before stating our QBA framework, we introduce the multicast round. In a multicast round with *n* players, there is a primary, and the others are backups. The primary multicasts his or her message to the backups by the following operation, as shown in Fig. 1. One of the backups is selected as the forwarder, and the other unchosen backups become verifiers. The primary, the forwarder, and one of the verifiers perform a three-party QDS to transmit the message. QDS is divided into two stages—distribution and messaging stage. The distribution stage is to distribute correlated quantum keys to the players. The messaging stage uses some classical operations and quantum keys to complete digital signatures. The messaging stage contains three steps: signing, forwarding, and verification. The primary signs the message and then sends the message and corresponding signature to the forwarder. After that, the forwarder will forward the message and signature to the verifier. Only when both the forwarder and verifier accept the signature, the signing is successful, i.e., the primary cannot deny the fact that she signed the message (nonrepudiation), and the message cannot be forged by others including the forwarder (unforgeability) (see Materials and Methods for details). For a chosen forwarder, the verifiers take turns participating in such three-party QDS. The above process will be repeated until all backups have acted as the forwarder one time. In the end, each backup records a list of *n* − 1 messages, consisting of one message directly received from the primary and *n* − 2 messages forwarded by other backups. We call this list of messages broadcasting list in the later. Note that a complete multicast round consists of three steps: (1) sign and multicast, (2) forward, and (3) verify and record.

Generally, our QBA framework consists of two phases, namely, broadcasting phase and gathering phase. Suppose there is a system of total *N* players including *f* malicious ones. The commanding general (initial primary) is denoted as *S*, and the lieutenants are denoted as *R_i_*, for *i* = 1, 2, ⋯, *N* − 1. The flow chart of the two phases is shown in Table 2. The broadcasting phase is designed for *R_i_* to exchange the message received from *S* with each other, and the gathering phase is designed for *R_i_* to deduce the original message of *S* according to the information gathered by themselves.

**Table 2.** Protocol definition

**Broadcasting phase**
The broadcasting phase begins with *d* = 1 and ends with *d* = *f*. We consider a general case $MR_{\zeta }^{d}$ (*d*=1, 2, 3, ⋯, *f*):
**1. Sign and multicast** The primary signs and then multicasts the message $m_{\zeta }^{d}$ to the *N–d* backups via QDS as shown in Fig. 1.
**2. Consistency check** If *d* = 1, there is no consistency check and the players will skip to step 3. If 1 < *d* ≤ f, upon receiving the message from the primary, forwarder *R_j_* checks the consistency between it and the message that he received from the primary at the previous depth *d* − 1. (*R_j_* visits the players who do not appear in route ζ.) If consistency check is passed, perform step 3. Otherwise, he requests that this primary perform step 1 again until he receives a consistent message.
**3. Forward** *R_j_* forwards the message to verifiers *R_k_* (*R*_k_ visit the players except *R_j_* and those who have appeared in route ζ).
**4. Verify and record** Forwarder *R_j_* and verifier *R_k_* verify the message and corresponding signature. When both of them accept, the signature is successful and they add this valid message $m_{\varsigma }^{d}$ to their own broadcasting lists $B_{\varsigma }^{d,R_{j}}$ and $B_{\varsigma }^{d,R_{k}}$, respectively.
**5. Recursion** The forwarder *R_j_* acts as the primary of ${\mathrm{MR}}_{\varsigma \to R_{j}}^{d+1}$, and then repeat the above four steps. The recursion process ends up when *d* = *f*.
**Gathering phase**
For the lieutenants *R_i_* (*i* = 1, 2, ..., *N* − 1):
**1. Input** In the bottom layer *d* = *f*, *R_i_* obtains the initial gathering lists $G_{\varsigma }^{f,R_{i}}=B_{\varsigma }^{f,R_{i}}$.
**2. Recursion** When 1 ≤ *d* < *f*, the gathering lists at the corresponding depth and route are $G_{\varsigma }^{f,R_{i}}=\cup_{R_{p}}\left\{m_{\varsigma \to R_{p}}^{d+1,R_{i}}\right\},$ where $m_{\varsigma \to R_{p}}^{d+1,R_{i}}=\text{majority}\left(G_{\varsigma \to R_{p}}^{d+1,R_{i}}\right)$ and *R_p_* visits all players except those who have appeared in the route ζ.
**3. Output** $m_{S}^{1,R_{i}}$ = majority $\left(G_{S}^{1,R_{i}}\right)$.

*Broadcasting phase.* The broadcasting phase consists of successive multicast rounds. For clarity, we denote the multicast round as $MR_{\zeta }^{d}$, where *ζ* represents the route of delivering the message and *d* is the depth of the multicast round. The first multicast round started by the commanding general *S* is denoted as $MR_{S}^{1}$. In $MR_{S}^{1}$, *S* signs and then multicasts his message $m_{S}^{1}$ to all the lieutenants *R_i_*. In the multicast round of next depth, $MR_{S\to R_{i}}^{2}$, *R_i_* acts as a primary, and then signs and multicasts the message $m_{S\to R_{i}}^{2}$, which is he received from *S*, to the other lieutenants. The process will be repeated until *d* = *f*. We denote a list for the lieutenants *R_i_* to record the messages received by him in $MR_{\zeta }^{d}$ as aforementioned broadcasting list $B_{\zeta }^{d,R_{i}}$.

In the broadcasting phase, the consistency check occurs between step 1 and step 3. Consider a general case: In $MR_{\zeta }^{d}$, the primary signs and multicasts the message $m_{\zeta }^{d}$ to the backups, assuming that *R_j_* acts as the forwarder and *R_k_* acts as a verifier. In the multicast round at next depth *d* + 1, $MR_{\zeta \to R_{j}}^{d+1}$, *R_j_* will act as a primary and *R_k_* will act as the forwarder. The messages *R_j_* delivers to *R_k_* in the two rounds, $MR_{\zeta }^{d}$ and $MR_{\zeta \to R_{j}}^{d+1}$, must be consistent, because *R_k_* can check the consistency of the two messages. If the two messages are inconsistent, *R_k_* will reject them and ask *R_j_* to repeat the process until the two messages are consistent.

*Gathering phase.* The deterministic function we used in the gathering phase is called the majority function. It outputs the value of the majority element in the input set (see Materials and Methods). In $MR_{\zeta }^{d}$, the gathering list held by the lieutenant *R_i_*, denoted as $G_{\zeta }^{d,R_{i}}$, is used for *R_i_* to deduce the message delivered by the primary of $MR_{\zeta }^{d}$. In the bottom layer *d* = *f*, *R_i_* directly sets his or her own gathering list to $G_{\zeta }^{f,R_{i}}=B_{\zeta }^{f,R_{i}}$ and outputs $m_{\zeta }^{f,R_{i}}=\text{majority}\left(G_{\zeta }^{f,R_{i}}\right)$. Then, $m_{\zeta }^{f,R_{i}}$ becomes an element of the gathering list of *d* = *f* − 1. Considering general case where 1 ≤ *d* < *f*, all elements of *R_i_*’s gathering list $G_{\zeta }^{d,R_{i}}$ are deduced from the lists $G_{\zeta \to R_{p}}^{d+1,R_{i}}$ in multicast round $MR_{\zeta \to R_{p}}^{d+1}$ (*R_p_* visits all players who do not appear in route *ζ*). When *p* = *i*, this element is directly set as the message that *R_i_* directly received from the primary in $MR_{\zeta }^{d}$. With the recursive process, the gathering phase ends up when *d* = 1, and then *R_i_* outputs $m_{S}^{1,R_{i}}=\text{majority}\left(G_{S}^{1,R_{i}}\right)$ as the final decision. Note that the broadcasting lists record the messages that are the lieutenants themselves actually received during the broadcast phase, and the gathering lists record the messages that are the lieutenants deduced according to the information of the previous depth. Only when *d* = *f*, the gathering lists are the same as the broadcasting lists, i.e., $G_{\zeta }^{f,R_{i}}=B_{\zeta }^{f,R_{i}}$.

### Experimental implementation

We show proof-of-principle experimental implementation of our QBA framework for reaching consensus on a decentralized digital ledger, one of the most important application of blockchain. The digital ledger is a 1.10-MB document that is a virtual transaction including time, clients, merchants, commodity, and the amount. It is converted into a binary string of bits. We denote the correct message as *m1*, and the incorrect messages as *m2*, *m3*, and so on.

To show a high degree of adaptability of our work, we implement three-party consensus with single-bit GC01-QDS [43], one-time universal_2_ hashing (OTUH) QDS [56], and OTUH-QDS without perfect keys [57], respectively (see Supplementary Materials Section B for details). In addition, we utilize OTUH-QDS to realize the five-party consensus. The key idea of single-bit GC01-QDS is to first generate two pairs of raw quantum keys and then exchange half of the Bob’s and Charlie’s quantum keys with each other, which is called symmetrization step, to construct the correlation. On the contrary, OTUH-QDS is to first construct the three-party correlation *X_a_* = *X_b_* ⊕ *X_c_* and *Y_a_* = *Y_b_* ⊕ *Y_c_* among the quantum keys of Alice (signer), Bob (forwarder), and Charlie (verifier). Alice signs the message with *X_a_* and *Y_a_*, and then Bob and Charlie exchange their keys to complete the verification. These correlated raw quantum keys can be achieved by any quantum key generation process [58–67]. Here, we utilize four-intensity decoy-state BB84 key generation process for the three QDS protocols [58].

**Fig. 2.**
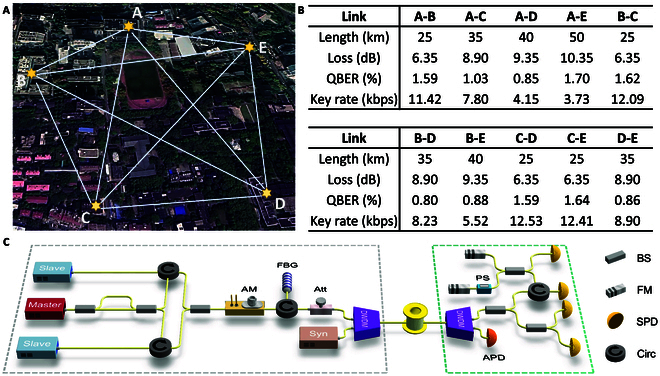
Experimental implementation. (A) The five players bring their own pre-distributed correlated quantum keys to five different buildings and then perform the classical operations of the messaging stage. A, *S*; B, *R*_1_; C, *R*_2_; D, *R*_4_; and E, *R*_5_. In the messaging stage, the messages and corresponding signatures can be transmitted via authenticated classical channel. (B) Main parameters of BB84 key generation in the laboratory. QBER, quantum bit error rate. (C) Experimental setup of the four-intensity decoy-state quantum key generation system with a time-phase encoding. We take nodes A and B as an example. A uses a master laser, two slave lasers, and an asymmetric interferometer to prepare optical pulses in the Z and X bases. An intensity modulator is used for the decoy-state modulation. Before passing through a set of filters, a monitor and an attenuator are utilized to regulate the photon number per pulse. B uses a biased beam splitter for the passive basis detection. The pulses either go directly to the time detector or pass through an asymmetric interferometer. A synchronization signal is distributed from node A to node B through a wavelength division multiplexed quantum channel. BS, beam splitter; Circ, circulator; IM, intensity modulator; FBG, fiber Bragg grating; Att, attenuator; DWDM, dense wavelength division multiplexer; FM, Faraday mirror; PS, phase shifter; SPD, single-photon detector.

There are five independent players *S* and *R_i_* (*i* = 1, 2, 3, 4). The correlated quantum keys of different pairwise players are pre-distributed in the laboratory via fiber spool, i.e., the distribution stage of QDS is completed in the laboratory. They do not disclose any information of their own quantum keys to others, and then bring the keys to five different buildings in Fig. 2 to simulate the real-life situation where the users are geographically separated, and then use these quantum keys to complete digital signatures, i.e., classical operations of the messaging stage are performed in the real locations. Note that, to simplify the proof-of-principle experiment, we employ the above method due to the immaturity of real-life multi-node quantum networks. It is anticipated that as quantum networks progress in maturity, enabling their widespread deployment and utilization, our QBA framework can be seamlessly integrated into practical quantum networks without the necessity of laboratory-based quantum key preparation.

**Fig. 3.**
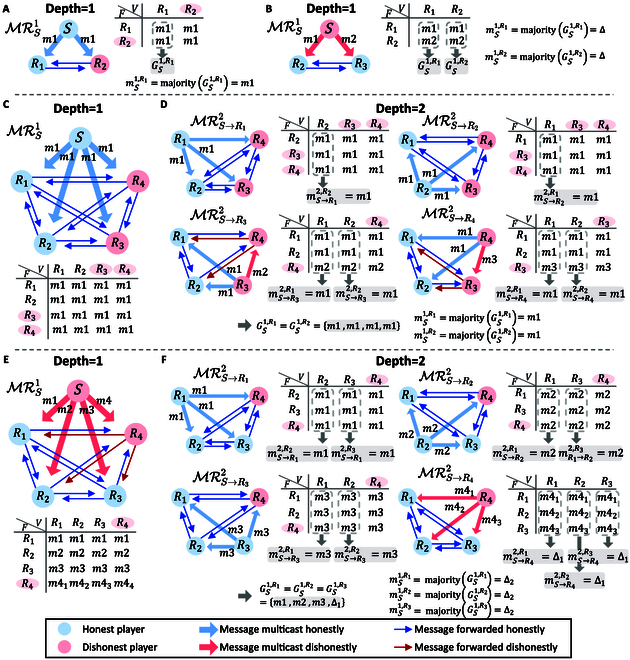
Experimental results for three-party and five-party consensus. We use “F” to represent “forwarder” and “V” to represent “verifier” in the tables of lists. Each column of a table is a broadcasting list for the corresponding player. In the bottom layer *d* = *f*, *R_i_* sets his or her gathering list as $G_{\zeta }^{f,R_{i}}=B_{\zeta }^{f,R_{i}}$ and performs the gathering phase to deduce the final output. (A) Multicast round at *d* = 1 in three-party consensus with an honest primary. (B) Multicast round at *d* = 1 in three-party consensus with a dishonest primary. Δ = majority(*m*1, *m*2). (C and D) Multicast rounds at *d* = 1 (*d* = 2) in five-party consensus with an honest initial primary. (E and F) Multicast rounds at *d* = 1 (*d* = 2) in five-party consensus with a dishonest initial primary. Δ_1_ = majority(*m*4_1_, *m*4_2_, *m*4_3_) and Δ_2_ = majority(*m*1, *m*2, *m*3, Δ_1_).

*S*, *R*_1_, and *R*_2_ perform three-party consensus, and all the five players perform five-party consensus. According to IC_1_ and IC_2_, we consider whether *S* is honest or not. Here, we exemplify the three-party and five-party consensus in Fig. 3. Moreover, we show the consensus rates of our QBA framework when adopting these three QDS in Table 3. OTUH-QDS, which can sign a multi-bit message each time, leads to much higher efficiency of QBA framework that the system can reach consensus 11.95 times per second, while single-bit GC01-QDS only reaches 4.5 × 10^−8^ times consensus per second under the same security parameter (see Supplementary Materials Section D for calculation details).

**Table 3.** Consensus rates of our QBA framework adopting different QDS in the three-party consensus. The agreement rate, *CR*, is defined as the number of times a system can reach consensus per second. It can be expressed as $\mathrm{CR}=\frac{\mathrm{SR}}{C}$, where *C* is the communication complexity of the system and *SR* is the signature rate of adopted QDS (see Materials and Methods)

Different kinds of QDS	Consensus rate
GC01-QDS [43]	4.5 × 10^−8^
OTUH-QDS [56]	11.95
OTUH-QDS without perfect keys [57]	6.12

**(a)** The commanding general *S* is honest in three-party consensus. There is only one malicious player *R*_2_ and thus only one layer *d* = 1 in the three-party consensus. In $MR_{S}^{1}$, *S* sends correct message *m1* via multicasting. *R*_1_ records *m1* when he acts as a forwarder, and records *m1* received from *R*_2_ when he acts as a verifier. The malicious *R*_2_ must honestly forward *m1* when he acts as a forwarder due to the unforgeability of QDS. Hence, as shown in Fig. 3A, the gathering list of honest *R*_1_, which is also the broadcasting list, is $G_{S}^{1,R_{1}}=B_{S}^{1,R_{1}}=\left\{m1,m1\right\}$. The final output of *R*_1_ is $m_{S}^{1,R_{1}}=\text{majority}\left(G_{S}^{1,R_{1}}\right)=m1$, which is consistent with the message sent by honest *S*. That satisfies IC_1_.

**(b)** The commanding general *S* is dishonest in three-party consensus. There is only one malicious player *S*. In $MR_{S}^{1}$, *S* sends conflicting messages *m1* and *m2* to honest *R*_1_ and *R*_2_, respectively. *R*_1_ records *m1* when he acts as a forwarder, and records *m2* received from *R*_2_ when he acts as a verifier. *R*_2_ records *m2* when he acts as a forwarder, and records *m1* received from *R*_1_ when he acts as a verifier. Hence, as shown in Fig. 3B, the gathering list of honest *R*_1_, which is also the broadcasting list, is $G_{S}^{1,R_{1}}=B_{S}^{1,R_{1}}=\left\{m1,m2\right\}$. The gathering list of honest *R*_2_ is $G_{S}^{1,R_{2}}=B_{S}^{1,R_{2}}=\left\{m1,m2\right\}$, which is the same as that of *R*_1_. The final outputs of *R*_1_ and *R*_2_ are both $m_{S}^{1,R_{1}}=m_{S}^{1,R_{2}}=\text{majority}\left(\left\{m1,m2\right\}\right)=\Delta$. Although the dishonest primary *S* sends conflicting messages, honest *R*_1_ and *R*_2_ obtain the same output Δ. That satisfies IC_2_.

**(c)** The commanding general *S* is honest in five-party consensus. There are two malicious players *R*_3_ and *R*_4_ (*f* = 2), and there are two layers *d* = 1 and *d* = 2 in five-party consensus.

*Broadcasting phase*. At depth *d* = 1, in $MR_{S}^{1}$, *S* broadcasts the correct message *m*_1_ via multicast process. In $MR_{S}^{1}$, every one must forward the correct message *m*_1_ to honest players because *S* is honest and there are two honest players in QDS. Therefore, the broadcasting lists of honest *R*_1_ and *R*_2_ are both $B_{S}^{1,R_{1}}=B_{S}^{1,R_{2}}=\left\{m1,m1,m1,m1\right\}$ (see Fig. 3C).

The results of the multicast processes at depth *d* = 2 are shown in Fig. 3D. In $MR_{S\to R_{1}}^{2}$, because of the honest primary *R*_1_ and Lemma 1 (see Materials and Methods for two lemmas), every one must forward the correct message *m*_1_, and the broadcasting list of honest *R*_2_ is $B_{S\to R_{1}}^{2,R_{2}}=\left\{m1,m1,m1\right\}$. In $MR_{S\to R_{2}}^{2}$, because of the honest primary *R*_2_ and Lemma 1, every one must forward the correct message *m1*, and the broadcasting list of honest *R*_1_ is $B_{S\to R_{2}}^{2,R_{1}}=\left\{m1,m1,m1\right\}$. In $MR_{S\to R_{3}}^{2}$, because of the dishonest primary *R*_3_ and Lemma 2, only when dishonest *R*_3_ and *R*_4_ collude together can *R*_4_ successfully forward the conflicting message *m2* to honest players. The broadcasting lists of honest *R*_1_ and *R*_2_ are both $B_{S\to R_{3}}^{2,R_{1}}=B_{S\to R_{3}}^{2,R_{2}}=\left\{m1,m1,m2\right\}$. Similar to the analysis of $MR_{S\to R_{3}}^{2}$, only when dishonest *R*_3_ and *R*_4_ collude together, *R*_4_ can successfully forward the conflicting message *m*_2_ to honest players. The broadcasting lists of honest *R*_1_ and *R*_2_ are both $B_{S\to R_{3}}^{2,R_{1}}=B_{S\to R_{3}}^{2,R_{2}}=\left\{m1,m1,m3\right\}$.

*Gathering phase.* At the bottom depth *d* = 2, each player obtains the initial gathering lists $G_{\zeta }^{2,R_{i}}=B_{\zeta }^{2,R_{i}}$.

For *R*_1_ at depth *d* = 2,

$$\begin{array}{ll} G_{S\to R_{2}}^{2,R_{1}} & =B_{S\to R_{2}}^{2,R_{1}}=\left\{m1,m1,m1\right\},\\ G_{S\to R_{3}}^{2,R_{1}} & =B_{S\to R_{3}}^{2,R_{1}}=\left\{m1,m1,m2\right\},\\ G_{S\to R_{4}}^{2,R_{1}} & =B_{S\to R_{4}}^{2,R_{1}}=\left\{m1,m1,m3\right\},\\ m_{S\to R_{1}}^{2,R_{1}} & =m1,\;\\ m_{S\to R_{2}}^{2,R_{1}} & =\text{majority}\left(G_{S\to R_{2}}^{2,R_{1}}\right)=m1,\\ m_{S\to R_{3}}^{2,R_{1}} & =\text{majority}\left(G_{S\to R_{3}}^{2,R_{1}}\right)=m1,\\ m_{S\to R_{4}}^{2,R_{1}} & =\text{majority}\left(G_{S\to R_{4}}^{2,R_{1}}\right)=m1, \end{array}$$ (1)

where the message $m_{S\to R_{1}}^{2,R_{1}}$ is directly received from *S* when *R*_1_ acts as the forwarder. *R*_1_ deduced that $m_{S\to R_{2}}^{2,R_{1}}$ is the message that *R*_2_ received from *S*, $m_{S\to R_{3}}^{2,R_{1}}$ is the message that *R*_3_ received from *S*, and $m_{S\to R_{4}}^{2,R_{1}}$ is the message that *R*_4_ received from *S*. Then, $m_{S\to R_{1}}^{2,R_{1}}$, $m_{S\to R_{2}}^{2,R_{1}}$, $m_{S\to R_{3}}^{2,R_{1}}$, and $m_{S\to R_{4}}^{2,R_{1}}$ constitute the gathering list of *R*_1_ at depth *d* = 1, where $G_{S}^{1,R_{1}}=\left\{m_{S\to R_{1}}^{2,R_{1}},m_{S\to R_{2}}^{2,R_{1}},m_{S\to R_{3}}^{2,R_{1}},m_{S\to R_{4}}^{2,R_{1}}\right\}=\left\{m1,m1,m1,m1\right\}$. Thus, at depth *d* = 1, *R*_1_ obtains the final output

$$\begin{array}{ll} m_{S}^{1,R_{1}} & =\text{majority}\left(G_{S}^{1,R_{1}}\right)\;\\ & =\text{majority}\left(\left\{m1,m1,m1,m1\right\}\right)\;\\ & =m1.\; \end{array}$$ (2)

For *R*_2_ at depth *d* = 2,

$$\begin{array}{cc} G_{S\to R_{1}}^{2,R_{2}} & =B_{S\to R_{1}}^{2,R_{2}}=\left\{m1,m1,m1\right\},\\ G_{S\to R_{3}}^{2,R_{2}} & =B_{S\to R_{3}}^{2,R_{2}}=\left\{m1,m1,m2\right\},\\ G_{S\to R_{4}}^{2,R_{2}} & =B_{S\to R_{4}}^{2,R_{2}}=\left\{m1,m1,m3\right\},\\ m_{S\to R_{1}}^{2,R_{2}} & =\text{majority}\left(G_{S\to R_{1}}^{2,R_{2}}\right)=m1,\\ m_{S\to R_{2}}^{2,R_{2}} & =m1,\;\\ m_{S\to R_{3}}^{2,R_{2}} & =\text{majority}\left(G_{S\to R_{3}}^{2,R_{2}}\right)=m1,\\ m_{S\to R_{4}}^{2,R_{2}} & =\text{majority}\left(G_{S\to R_{4}}^{2,R_{2}}\right)=m1, \end{array}$$ (3)

where the message $m_{S\to R_{2}}^{2,R_{2}}$ is directly received from *S* when *R*_2_ acts as the forwarder. *R*_2_ deduced that $m_{S\to R_{1}}^{2,R_{2}}$ is the message that *R*_1_ received from *S*, $m_{S\to R_{3}}^{2,R_{2}}$ is the message that *R*_3_ received from *S*, and $m_{S\to R_{4}}^{2,R_{2}}$ is the message that *R*_4_ received from *S*. Then, $m_{S\to R_{1}}^{2,R_{2}}$, $m_{S\to R_{2}}^{2,R_{2}}$, $m_{S\to R_{3}}^{2,R_{2}}$, and $m_{S\to R_{4}}^{2,R_{2}}$ constitute the gathering list of *R*_2_ at depth *d* = 1, where $G_{S}^{1,R_{2}}=\left\{m_{S\to R_{1}}^{2,R_{2}},m_{S\to R_{2}}^{2,R_{2}},m_{S\to R_{3}}^{2,R_{2}},m_{S\to R_{4}}^{2,R_{2}}\right\}=\left\{m1,m1,m1,m1\right\}$. Thus, at depth *d* = 1, *R*_2_ obtains the final output

$$\begin{array}{ll} m_{S}^{1,R_{2}} & =\text{majority}\left(G_{S}^{1,R_{2}}\right)\;\\ & =\text{majority}\left(\left\{m1,m1,m1,m1\right\}\right)\;\\ & =m1.\; \end{array}$$ (4)

The final outputs of *R*_1_ and *R*_2_ are both $m_{S}^{1,R_{1}}=m_{S}^{1,R_{2}}=m_{S}^{1,R_{1}}=m_{S}^{1,R_{2}}=\text{majority}\left(\left\{m_{1},m_{1},m_{1}m_{1}\right\}\right)=m_{1}$, which is consistent with the initial message sent by the honest primary *S*. This result satisfies IC_1_.

**(d)** The commanding general *S* is dishonest in five-party consensus. There are two malicious players *S* and *R*_4_ (*f* = 2), and there are two layers *d* = 1 and *d* = 2 in five-party consensus.

*Broadcasting phase.* At depth *d* = 1, in $MR_{S}^{1}$, the dishonest primary *S* can broadcast the different messages *m1*, *m2*, and *m3* to honest *R*_1_, *R*_2_, and *R*_3_, respectively, via multicast process. Additionally, due to Lemma 2, malicious *S* and *R*_4_ can collude together and then *R*_4_ can deliberately forward different messages *m*4_1_, *m*4_2_, and *m*4_3_ to honest *R*_1_, *R*_2_, and *R*_3_, respectively. Therefore, the broadcasting lists of honest *R*_1_, *R*_2_, and *R*_3_ are $B_{S}^{1,R_{1}}=\left\{m1,m2,m3,m4_{1}\right\}$, $B_{S}^{1,R_{2}}=\left\{m1,m2,m3,m4_{2}\right\}$, and $=B_{S}^{1,R_{3}}=\left\{m1,m2,m3,m4_{3}\right\}$, respectively (see Fig. 3E).

The results of the multicast processes at depth *d* = 2 are shown in Fig. 3F. In $MR_{S\to R_{1}}^{2}$, because of the honest primary *R*_1_ and Lemma 1, every one must forward *R*_1_’s message *m*_1_, and the broadcasting lists of honest *R*_2_ and *R*_3_ are both $B_{S\to R_{1}}^{2,R_{2}}=B_{S\to R_{1}}^{2,R_{3}}=\left\{m1,m1,m1\right\}$. In $MR_{S\to R_{2}}^{2}$, because of the honest primary *R*_2_ and Lemma 1, every one must forward *R*_1_’s message *m2*, and the broadcasting lists of honest *R*_1_ and *R*_3_ are both $B_{S\to R_{2}}^{2,R_{1}}=B_{S\to R_{2}}^{2,R_{3}}=\left\{m2,m2,m2\right\}$. In $MR_{S\to R_{3}}^{2}$, because of the honest primary *R*_2_ and Lemma 1, every one must forward *R*_3_’s message *m3*, and the broadcasting lists of honest *R*_1_ and *R*_2_ are both $B_{S\to R_{3}}^{2,R_{1}}=B_{S\to R_{3}}^{2,R_{2}}=\left\{m3,m3,m3\right\}$. In $MR_{S\to R_{4}}^{2}$, because the dishonest primary *R*_4_ can successfully broadcast the conflicting messages *m*4_1_, *m*4_2_, and *m*4_3_ to honest players *R*_1_, *R*_2_, and *R*_3_, respectively. The broadcasting lists of honest *R*_1_, *R*_2_, and *R*_3_ are all $B_{S\to R_{4}}^{2,R_{1}}=B_{S\to R_{4}}^{2,R_{2}}=B_{S\to R_{4}}^{2,R_{3}}=\left\{m4_{1},m4_{2},m4_{3}\right\}$.

*Gathering phase.* At the bottom depth *d* = 2, each player obtains the initial gathering lists $G_{\zeta }^{2,R_{i}}=B_{\zeta }^{2,R_{i}}$.

For *R*_1_ at depth *d* = 2,

$$\begin{array}{ll} G_{S\to R_{2}}^{2,R_{1}} & =B_{S\to R_{2}}^{2,R_{1}}=\left\{m2,m2,m2\right\},\;\\ G_{S\to R_{3}}^{2,R_{1}} & =B_{S\to R_{3}}^{2,R_{1}}=\left\{m3,m3,m3\right\},\;\\ G_{S\to R_{4}}^{2,R_{1}} & =B_{S\to R_{4}}^{2,R_{1}}=\left\{m4_{1},m4_{2},m4_{3}\right\},\\ m_{S\to R_{1}}^{2,R_{1}} & =m1,\;\\ m_{S\to R_{2}}^{2,R_{1}} & =\text{majority}\left(G_{S\to R_{2}}^{2,R_{1}}\right)=m2,\;\\ m_{S\to R_{3}}^{2,R_{1}} & =\text{majority}\left(G_{S\to R_{3}}^{2,R_{1}}\right)=m3,\;\\ m_{S\to R_{4}}^{2,R_{1}} & =\text{majority}\left(G_{S\to R_{4}}^{2,R_{1}}\right)=\Delta_{1},\; \end{array}$$ (5)

where the message $m_{S\to R_{1}}^{2,R_{1}}$ is directly received from *S* when *R*_1_ acts as the forwarder. *R*_1_ deduced that $m_{S\to R_{2}}^{2,R_{1}}$ is the message that *R*_2_ received from *S*, $m_{S\to R_{3}}^{2,R_{1}}$ is the message that *R*_3_ received from *S*, and $m_{S\to R_{4}}^{2,R_{1}}$ is the message that *R*_4_ received from *S*. Then, $m_{S\to R_{1}}^{2,R_{1}}$, $m_{S\to R_{2}}^{2,R_{1}}$, $m_{S\to R_{3}}^{2,R_{1}}$, and $m_{S\to R_{4}}^{2,R_{1}}$ constitute the gathering list of *R*_1_ at depth *d* = 1, where $G_{S}^{1,R_{1}}=\left\{m_{S\to R_{1}}^{2,R_{1}},m_{S\to R_{2}}^{2,R_{1}},m_{S\to R_{3}}^{2,R_{1}},m_{S\to R_{4}}^{2,R_{1}}\right\}=\left\{m1,m2,m3,\Delta_{1}\right\}$. Thus, at depth *d* = 1, *R*_1_ obtains the final output

$$\begin{array}{ll} m_{S}^{1,R_{1}} & =\text{majority}\left(G_{S}^{1,R_{1}}\right)\;\\ & =\text{majority}\left(\left\{m1,m2,m3\Delta_{1}\right\}\right)\;\\ & =\Delta_{2}.\; \end{array}$$ (6)

For *R*_2_ at depth *d* = 2,

$$\begin{array}{ll} G_{S\to R_{1}}^{2,R_{2}} & =B_{S\to R_{1}}^{2,R_{2}}=\left\{m1,m1,m1\right\},\;\\ G_{S\to R_{3}}^{2,R_{2}} & =B_{S\to R_{3}}^{2,R_{2}}=\left\{m3,m3,m3\right\},\;\\ G_{S\to R_{4}}^{2,R_{2}} & =B_{S\to R_{4}}^{2,R_{2}}=\left\{m4_{1},m4_{2},m4_{3}\right\},\;\\ m_{S\to R_{1}}^{2,R_{2}} & =\text{majority}\left(G_{S\to R_{2}}^{2,R_{2}}\right)=m1,\;\\ m_{S\to R_{2}}^{2,R_{2}} & =m2,\;\\ m_{S\to R_{3}}^{2,R_{2}} & =\text{majority}\left(G_{S\to R_{3}}^{2,R_{2}}\right)=m3,\;\\ m_{S\to R_{4}}^{2,R_{2}} & =\text{majority}\left(G_{S\to R_{4}}^{2,R_{2}}\right)=\Delta_{1},\; \end{array}$$ (7)

where the message $m_{S\to R_{2}}^{2,R_{2}}$ is directly received from *S* when *R*_2_ acts as the forwarder. *R*_2_ deduced that $m_{S\to R_{1}}^{2,R_{2}}$ is the message that *R*_1_ received from *S*, $m_{S\to R_{3}}^{2,R_{2}}$ is the message that *R*_3_ received from *S*, and $m_{S\to R_{4}}^{2,R_{2}}$ is the message that *R*_4_ received from *S*. Then, $m_{S\to R_{1}}^{2,R_{2}}$, $m_{S\to R_{2}}^{2,R_{2}}$, $m_{S\to R_{3}}^{2,R_{2}}$, and $m_{S\to R_{4}}^{2,R_{2}}$ constitute the gathering list of *R*_2_ at depth *d* = 1, where $G_{S}^{1,R_{2}}=\left\{m_{S\to R_{1}}^{2,R_{2}},m_{S\to R_{2}}^{2,R_{2}},m_{S\to R_{3}}^{2,R_{2}},m_{S\to R_{4}}^{2,R_{2}}\right\}=\left\{m1,m2,m3,\Delta_{1}\right\}$. Thus, at depth *d* = 1, *R*_2_ obtains the final output

$$\begin{array}{ll} m_{S}^{1,R_{2}} & =\text{majority}\left(G_{S}^{1,R_{2}}\right)\;\\ & =\text{majority}\left(\left\{m1,m2,m3,\Delta_{1}\right\}\right)\;\\ & =\Delta_{2}.\; \end{array}$$ (8)

For *R*_3_ at depth *d* = 2,

$$\begin{array}{ll} G_{S\to R_{1}}^{2,R_{3}} & =B_{S\to R_{1}}^{2,R_{2}}=\left\{m1,m1,m1\right\},\;\\ G_{S\to R_{2}}^{2,R_{3}} & =B_{S\to R_{2}}^{2,R_{2}}=\left\{m2,m2,m2\right\},\;\\ G_{S\to R_{4}}^{2,R_{3}} & =B_{S\to R_{4}}^{2,R_{2}}=\left\{m4_{1},m4_{2},m4_{3}\right\},\;\\ m_{S\to R_{1}}^{2,R_{3}} & =\text{majority}\left(G_{S\to R_{2}}^{2,R_{3}}\right)=m1,\;\\ m_{S\to R_{2}}^{2,R_{3}} & =\text{majority}\left(G_{S\to R_{2}}^{2,R_{3}}\right)=m2,\;\\ m_{S\to R_{3}}^{2,R_{3}} & =m3,\;\\ m_{S\to R_{4}}^{2,R_{3}} & =\text{majority}\left(G_{S\to R_{4}}^{2,R_{3}}\right)=\Delta_{1},\; \end{array}$$ (9)

where the message $m_{S\to R_{2}}^{2,R_{2}}$ is directly received from *S* when *R*_3_ acts as the forwarder. *R*_3_ deduced that $m_{S\to R_{1}}^{2,R_{3}}$ is the message that *R*_1_ received from *S*, $m_{S\to R_{2}}^{2,R_{3}}$ is the message that *R*_2_ received from *S*, and $m_{S\to R_{4}}^{2,R_{3}}$ is the message that *R*_4_ received from *S*. Then, $m_{S\to R_{1}}^{2,R_{3}}$, $m_{S\to R_{2}}^{2,R_{3}}$, $m_{S\to R_{3}}^{2,R_{3}}$, and $m_{S\to R_{4}}^{2,R_{3}}$ constitute the gathering list of *R*_3_ at depth *d* = 1, where $G_{S}^{1,R_{3}}=\left\{m_{S\to R_{1}}^{2,R_{3}},m_{S\to R_{2}}^{2,R_{3}},m_{S\to R_{3}}^{2,R_{3}},m_{S\to R_{4}}^{2,R_{3}}\right\}=\left\{m1,m2,m3,\Delta_{1}\right\}$. Thus, at depth *d* = 1, *R*_3_ obtains the final output

$$\begin{array}{ll} m_{S}^{1,R_{3}} & =\text{majority}\left(G_{S}^{1,R_{3}}\right)\;\\ & =\text{majority}\left(\left\{m1,m2,m3,\Delta_{1}\right\}\right)\;\\ & =\Delta_{2}.\; \end{array}$$ (10)

The final outputs of honest *R*_1_, *R*_2_, and *R*_3_ are all $m_{S}^{1,R_{1}}=m_{S}^{1,R_{2}}=$$m_{S}^{1,R_{1}}=m_{S}^{1,R_{2}}=$$m_{S}^{1,R_{3}}=\Delta_{2}$. Although the dishonest primary *S* sends conflicting messages, the honest players *R*_1_, *R*_2_, and *R*_3_ obtain the same output Δ_2_. This result satisfies IC_2_.

In conclusion, our experimental results show that in real three-party and five-party consensus, our protocol can not only satisfy the two original Byzantine conditions IC_1_ and IC_2_ but also achieve the fault tolerance of *N* ≥ 2*f* + 1, which breaks the 1/3 fault-tolerance lower bound.

## Discussion

The 1/3 fault-tolerance bound cannot be beaten for any arbitrary pairwise communication [10–13]; not even quantum channels can help solve this problem. If the nodes of a system are linked by the channels that are independent of each other, the bound is unable to be beaten. Intriguingly, when quantum entanglement is introduced into the system, it is possible to surpass this bound because quantum entanglement provides the correlation and removes the independence [26]. Although detectable QBA framework is designed according to multi-particle entanglement [26–32], they cannot extend to more than three participants and unavoidably weaken the Byzantine conditions, because multi-particle entangled states are very hard to prepare and maintain, and these protocols do not fully utilize the correlation to protect the unforgeability and nonrepudiation that leads to a certain probability of failure.

Is quantum entanglement necessary or can weaker multiparty correlation work in Byzantine agreement? Fortunately, QDS is a useful tool for solving this problem due to its asymmetric relationship among three players. In addition, three-party QDS is naturally decentralized due to its structure without a fully trusted third party. The two essential properties of QDS, unforgeability and nonrepudiation, effectively curtail the malevolent activities of malicious players within the system, preventing them from deliberately delivering conflicting messages. Asymmetric relationship of QDS makes the channels no longer independent of each other. Consequently, our protocol can break the 1/3 fault-tolerance bound.

Note that the most important thing to break the fault-tolerance bound is to provide a decentralized multiparty correlation to remove the independence of pairwise channels. Intriguingly, quantum entanglement and asymmetric relationship of QDS both satisfy the above requirement. In addition, if we can find a three-party information-theoretically secure classical digital signature scheme that has the same decentralized structure as QDS [68], our framework can also break the bound. However, up to now, we do not find classical correlation greater than QDS without any additional assumptions because these classical schemes require extra assumptions such as the existence of a trusted third party and authenticated broadcast channels, which disobeys decentralization of Byzantine agreement [69]. By bridging two prominent research themes, the Byzantine agreement and QDS, our work paves the way for practical quantum blockchain and quantum consensus networks.

In the end, we want to highlight that although our QBA framework utilizes QDS, it is still unable to surpass the famous blockchain trilemma (see Materials and Methods).The blockchain trilemma highlights the intricate balance required among three fundamental attributes: decentralization, security, and scalability. Within our QBA framework, we have achieved notable success in security, surpassing the 1/3 fault-tolerance bound while maintaining information-theoretical security. Moreover, our approach has full decentralization. However, it falls short in scalability, exhibiting exponential communication complexity, as shown in Eq. 11. Consequently, our research remains bound by the constraints posed by the blockchain trilemma. Intriguing inquiries linger as we ponder the blockchain trilemma’s resilience to quantum resources. Is it an irrefutable theorem or an assailable postulate? The possibility of quantum resources challenging the blockchain trilemma beckons us toward further scholarly exploration.

## Materials and Methods

### Quantum digital signatures

QDSs with information-theoretical security have two major properties, nonrepudiation and unforgeability. They are all divided into the two stages, distribution stage and messaging stage. The distribution stage is to distribute the correlated raw quantum keys of Alice–Bob and Alice–Charlie for the messaging stage. The correlated quantum keys can be achieved by some classical operations, such as symmetrization step used in CV-QDS [49], BB84 GC01-QDS [43], and MDI-QDS [45,51], test bits used in SARG04-QDS [52,54,55], and secret sharing used in OTUH-QDS [56,57]. The messaging stage is to complete the digital signature to determine whether it is successful or not.

The brief process of messaging stage can be described as follows. Alice is a “signer.” Bob is a “forwarder.” Charlie is a “verifier.” Alice signs a message with her quantum keys and then transmits the message and corresponding signature to Bob. Bob forwards the message and corresponding signature to the verifier Charlie. Then, Bob and Charlie will check the message and corresponding signature, respectively. The process of QDS is successful when and only when both Bob and Charlie accept the message and the corresponding signature. The signature rate, *SR*, is defined as the number of times the players can perform the QDS per second.

*Nonrepudiation.* Nonrepudiation refers to a situation in which the signer cannot successfully dispute the authorship of his signature. This means that Alice cannot deny the fact that she signed the message if the signature is accepted by both Bob and Charlie.

*Unforgeability.* Unforgeability refers to a situation in which no one can forge a message and its corresponding signature. This means that if Bob forwards a forged message and signature, it will be impossible for him to successfully make Charlie accept the forged message and signature.

### Majority function

The majority function we apply in our protocol aims to output the element that appears most often for an input set. For example, when the input set is *M* = {*m*_1_, *m*_1_, *m*_1_, *m*_2_, *m*_2_}, the output will be majority(*M*) = *m*_1_. In a few cases, more than one element appears most frequently in the input set, and the systems that calculate the majority function on the input set are often deliberately biased toward one of them that we set initially. For example, when the input set is *M* = {*m*_1_, *m*_1_, *m*_1_, *m*_2_, *m*_2_, *m*_2_}, the output will be majority(*M*) = *m*_1_ (*m*_2_), which is determined by the biased output *m*_1_ (*m*_2_) that we set before calculation. Note that for the same input sets with different players, the majority function outputs the same value, which we will denote as Δ.

### Communication complexity and consensus rate

To measure the consumed resources, we define the number of times the QDS process is implemented to reach consensus as the communication complexity, denoted as *C*. The total communication complexity of our QBA protocol can be expressed by

$$\begin{array}{c} C=A_{N-1}^{2}+\left(N-1\right)A_{N-2}^{2}+\left(N-2\right)A_{N-3}^{2}\\ \;\;\;+\cdots +\left(N-1\right)\left(N-2\right)\cdots \left(N-f+1\right)A_{N-f}^{2}\\ \;\;\;\;=\sum_{m=0}^{f-1}A_{N-1}^{2+m}, \end{array}$$ (11)

where $A_{a}^{b}=\frac{a!}{\left(a-b\right)!}$ is *b*-permutations of *a*, *f* is the number of dishonest players, and *N* is the number of all players. Here, $A_{n-1}^{2+m}$ represents the communication complexity at depth *m* + 1. In reality, we need to perform $\left[\frac{N-1}{2}\right]$ recursions for a real-life consensus system with unknown *f*, where [*x*] is the greatest integer less than or equal to *x*.

Consider the simple case that the system uses the QDS protocol, which has the same signature rate, denoted as *SR*, in all the multicast rounds. We define the consensus rate of our QBA protocol as

$$\mathrm{CR}=\frac{\mathrm{SR}}{C}=\frac{\mathrm{SR}}{\sum_{m=0}^{f-1}‍A_{N-1}^{2+m}},$$ (12)

where *C* is the communication complexity of the system. *CR* is the important index to indicate the efficiency of QBA. To get the higher consensus rate, we need to adopt the QDS protocol that has the higher signature rate. We can find that as the total number of players and the number of malicious players increase, the communication complexity will increase, which leads to the decrease of the consensus rate.

### Two important lemmas in security analysis

In our protocol, the performance of honest (dishonest) players follows the same rule. Therefore, the players can be divided into two groups, the honest and dishonest. Also, the elements of a gathering list can be divided in the same way. Therefore, we can simplify the protocol with a perfect binary tree model where one tree node represents the set of multicast rounds with honest or dishonest primaries. The left (right) child tree node represents the multicast rounds with honest (dishonest) primaries of the next depth. In what follows, when we say a tree node is honest (dishonest), it means that the primaries in this tree node is honest (dishonest). We can obtain the important Lemma 1 and Lemma 2. The proofs for two lemmas and complete security analysis for our protocol can be found in Supplementary Materials Section C in detail.

Lemma 1: Suppose that B is a right child tree node of a parent node A who is honest, and C is the left child tree node of B. The messages delivered in C are consistent with those of A, which protects the consistency of the delivered messages.

Lemma 2: Suppose that B is a right child tree node of a parent node A who is honest, and E is the right child tree node of B. The message multicast in E can be inconsistent with those of A, which disrupts the consistency of the delivered messages.

### Experimental setting

The master laser generates phase-randomized 1.6-ns-wide laser pulses with a repetition rate of 100 MHz at 1550.12 nm. The system frequency is 100 MHZ, but due to the 400-ns dead time every 10 us, the effective frequency of optical pulse is 96 MHz. Two pairs of pulses with relative phases 0 and *π* at a 2-ns time delay generated by an asymmetric interferometer are injected into two slave lasers through the optical circulator, respectively. By controlling the trigger electrical signal of two slave lasers, Alice randomly prepares quantum states in the Z (time) and X (phase) bases by using 400-ps-wide slave laser pulses. The programmable delay chip with a 10-ps timing resolution is used to calibrate the time consistency. The spectral consistency is naturally satisfied because of the laser seeding technique [70]. A 50-GHz nominal bandwidth fiber Bragg grating is used to remove extra spurious emission and precompensate for the pulse broadening in the fiber transmission. The 2-ns-wide synchronization pulses with repetition rates of 100 kHz are transmitted via the quantum channel using multiplexed wavelength division. The intensities are set as *μ* = 0.40, *ν* = 0.20, *ω* = 0.4, and 0 with the corresponding probabilities *p_μ_* = 0.60, *p_ν_* = 0.20, *p_ω_* = 0.15, and *p*_0_ = 0.05, respectively. If trigger signal is not provided to the slave laser, the vacuum state is generated. The amplitude modulator generates two different intensities, and the intensity of *ω* is double that of *ν* (*ω* = 2*ν*) since it has two pulses in the X basis. At the receiving end, a 30:70 biased beam splitter is used to perform passive basis detection after a wavelength division demultiplexer. A probability of 30% is measured in the phase basis, and the probability of 70% is used to receive in the time basis. A Faraday–Michelson interferometer is used for the phase measurement, in which phase drift is compensated in real time by using the phase shifter. The total insertion losses of the time and phase bases are 4.25 and 8 dB, respectively. The efficiency of single-photon detectors is 20% at a 160 dark count per second. To decrease the after-pulse probability, we set the dead times to 10 μs for the links.

### Blockchain trilemma

Blockchain possesses three crucial attributes: decentralization, security, and scalability. Decentralization forms the fundamental core of blockchain technology, emphasizing its inherent nature. Security stands as a paramount concern in any blockchain system, while scalability presents a formidable challenge. However, the blockchain trilemma emerges from the inherent difficulty of achieving a harmonious balance among these three essential elements. Blockchains are often forced to make trade-offs that prevent them from achieving all the three aspects. Note that the trilemma is just a model to conceptualize the various challenges facing blockchain technology. There is no strict proof that the three aspects cannot be achieved. However, to date, there are no protocols able to break down the trilemma. The design needs to weaken the requirements for a certain feature.

## Data Availability

Data generated and analyzed during the current study are available from the corresponding author upon reasonable request.

## Supplementary Materials

Supplementary 1 Sections A to D Figs. S1 to S4
