# Supplementary material for: Beating the Fault-Tolerance Bound and Security Loopholes for Byzantine Agreement with a Quantum Solution
Source: Research (Wash D C). 2023 Nov 21;6:0272. doi: 10.34133/research.0272 (PMC11925315; doi:10.34133/research.0272)
Supplement: Supplementary 1 — Sections A to D Figs. S1 to S4 [file research.0272.f1.docx]

**Supplementary Materials**

**Beating the Fault-Tolerance Bound and Security Loopholes for Byzantine Agreement with a Quantum Solution**

Chen-Xun Wen$g^{1*}$, Rui-Qi Ga$o^{1*}$, Yu Bao^1^, Bing-Hong Li^1^, Wen-Bo Liu^1^, Yuan-Mei Xie^1^, Yu-Shuo Lu^1^, Hua-Lei Yi$n^{1,2\dagger}$, Zeng-Bing Che$n^{1\ddagger}$

^1^National Laboratory of Solid State Microstructures and School of Physics, Collaborative Innovation Center of Advanced Microstructures, Nanjing University, Nanjing 210093, China

^2^Department of Physics and Beijing Key Laboratory of Opto-electronic Functional Materials and Micro-nano Devices, Key Laboratory of Quantum State Construction and Manipulation (Ministry of Education), Renmin University of China, Beijing 100872, China

$*$ These authors contributed equally to this work

$\dagger$ Address correspondence to: [hlyin@nju.edu.cn](mailto:hlyin@nju.edu.cn)

‡Address correspondence to: [zbchen@nju.edu.cn](mailto:zbchen@nju.edu.cn)

This Supplemental Material has the following structure. First, in Section **A**, we supplement some pre-knowledge of blockchain and Byzantine consensus. Then, in Section **B**, we briefly introduce three different QDS we applied. In Section **C**, we provide a detailed security analysis using perfect binary tree mode. Finally, in Section **D**, we illustrate the experimental calculation details.

1. **Some pre-knowledge of Byzantine agreement**
2. **Blockchain**

Blockchain is a decentralized digital database technology that allows secure transactions between multiple parties without the need for intermediaries. It was first introduced in 2008 as the underlying technology for the cryptocurrency, Bitcoin. However, its potential applications have expanded beyond just cryptocurrencies. What makes blockchain unique is that it is a distributed system, which means that it is maintained by a network of nodes that are interested in maintaining it rather than a central authority. Every participant in the network holds a copy of the blockchain, and any changes to the database require consensus among the nodes. This makes it virtually impossible for a single entity to control or manipulate the blockchain.

The potential applications of blockchain are vast and include everything from cryptocurrency, financial transactions (digital ledgers), the Internet of Things and supply chain management to digital identity verification and voting systems. Its decentralized and secure nature makes it an attractive solution for businesses and organizations looking to streamline processes, increase efficiency, and reduce costs.

Blockchain includes many cryptography tasks, such as consensus, timestamp, identity authentication, privacy protection and so on. The most important one of them is the consensus problem, known as the Byzantine general problem, which is the research topic of our work. Our work does not aim to solve all the cryptography tasks of blockchain, and we focus on the core problem, Byzantine consensus.

1. **Byzantine general problem**

The Byzantine General Problem (also called Byzantine fault tolerance problem) is a classic computer science problem that deals with the challenge of coordinating a group of distributed and autonomous entities to reach a consensus in the presence of faulty or malicious actors~\cite{lamport1982byzantine}. In this problem, a group of Byzantine generals is camped outside a city and must coordinate their attack or retreat plans via messengers. However, some of the generals may be traitors who aim to sabotage the coordination, and messengers can be captured or corrupted during transmission, leading to false messages.

The challenge is to design a Byzantine agreement protocol that ensures that all loyal generals agree on a common plan of action, even in the presence of faulty or malicious actors. This problem has applications in distributed computing, cryptography, and especially blockchain technology. The Byzantine General Problem remains an active research topic in computer science and is considered a fundamental problem in distributed systems.

1. **Two necessary interactive consistency (IC) Byzantine condition**

Lamport et.al have proven that the Byzantine General Problem can be translated in a `commanding general-lieutenants' model, where the commanding general is randomly chosen from among all Byzantine generals and the others become lieutenants to reach consensus on the order of the commanding general~\cite{lamport1982byzantine}. A strict Byzantine agreement must satisfy the following two interactive consistency Byzantine conditions as follows. $IC_{1}$: All loyal lieutenants obey the same order. $IC_{2}$: Every loyal lieutenant obeys the order he or she sends if the commanding general is loyal. These two conditions emphasize two major concerns. When the commanding general is dishonest, all loyal players output consistent values. When the commanding general is honest, all loyal players output consistent and correct values. A strict Byzantine agreement must obey these two original conditions without adding any other assumptions. However, for detectable QBA protocols to achieve three-party consensus, an extra assumption is needed: there must be a certain probability that the protocol will fail. The players must discard the outcome when the protocol fails and perform the process again until the protocol succeeds. Therefore, all detectable QBA protocols are weaker versions of the Byzantine agreement.

1. **Quantum digital signatures**

Our QBA protocol can apply any kind of QDS to ensure unconditional security and better fault-tolerance performance.

1. **BB84-KGP GC01-QDS**

BB84-KGP GC01-QDS is a traditional single-bit QDS protocol proposed in 2016. In every round only one bit of message is signed. That is, possible message is $m$ = 0 or 1. In the distribution stage, bit correlations between Alice--Bob and Alice--Charlie are realized by BB84 key generation protocol (KGP). In the messaging stage users exchange partial of their keys and compare the mismatch rate to verify the signature. Here we introduce this protocol used in our quantum consensus experiment.

*Distribution stage---*

1. For $m$ = 0 or 1, Alice uses the BB84-KGP to generate four different keys of length $L$, $A_{B}^{0},A_{B}^{1},A_{C}^{0},A_{C}^{1}$, where the subscript $A$ and $B$ denotes she performed the KGP with Bob and Charlie, respectively, and the superscript denotes the future message to be signed, to be decided later by Alice. After BB84-KGP, Bob holds the length $L$ strings $K_{B}^{0},K_{B}^{1}$ and Charlie holds the length L strings $K_{C}^{0},\sim K_{C}^{1}$. The procedure of BB84-KGP is analogous to BB84-QKD, but error correction and privacy amplification steps are removed. The shared keys are correlated with limited mismatch and secrecy leakage.
2. Bob and Charlie symmetrize their keys by choosing half of the bit values in their $K_{B}^{m},K_{C}^{m}$ and sending them as well as the corresponding positions to each other using the Bob-Charlie secret classical channel. They will only keep the bits they did not forward and those received from the other participant. Their final symmetrized keys are denoted as $S_{B}^{m}$ and $S_{C}^{m}$. Bob (and Charlie) will keep a record of whether an element in $S_{B}^{m}$ ($S_{C}^{m}$) came directly from Alice or whether it was forwarded to him by Charlie (or Bob).

*Messaging stage---*

1. To send a signed one-bit message $m$, Alice sends $(m,Sig_{m})$ to the desired recipient (say Bob), where $sig_{m}=(A_{B}^{m},A_{C}^{m})$.
2. Bob checks whether $(m,Sig_{m}$) matches his $S_{B}^{m}$ and records the number of mismatches he finds. He separately checks the part of his key received directly from Alice and the part of the key received from Charlie. If there are fewer than $s_{a}(L/2)$ mismatches in both halves of the key, where $s_{a} <1/2$ is a small threshold determined by the parameters and the desired security level of the protocol, then Bob accepts the message.
3. To forward the message to Charlie, Bob forwards the pair $(m,Sig_{m})$ that he received from Alice.
4. Charlie tests for mismatches in the same way, but in order to protect against repudiation by Alice he uses a different threshold. Charlie accepts the forwarded message if the number of mismatches in both halves of his key is below $s_{v}(L/2)$ where $s_{v}$ is another threshold, with $0<s_{a}< s_{v}<1/2$.

*---Security of BB84-QDS*

The probability of a successful repudiation is

$$\varepsilon_{\mathrm{rep}} =2e^{-\left( s_{a}-s_{v} \right)^{2}L},$$

and that of a successful forgery is

$$\varepsilon_{\mathrm{for}} =2e^{-\frac{1}{4}\left( p_{e}-s_{a} \right)^{2}L} ,$$

where $p_{e}$ represents the unknown information of one bit in the string and can be bounded by parameters of BB84-KGP.

1. **One-time $\mathbf{universa}\mathbf{l}_{\mathbf{2}}$ hashing QDS**

We introduce the one-time $\mathrm{universa}l_{2}$ hashing (OTUH)-QDS we applied in our quantum consensus experiment, which utilizes secret sharing, one-time hashing and one-time pad to generate and verify signatures~\cite{yin2021experimental}.

*Distribution stage---*

Before executing the signature, Alice, Bob and Charlie all have two sets of keys, and , which satisfy the bit correlations ($p$ bits) and ($2p$ bits). The perfect bit correlation of three parties can be realized by using quantum communication, such as quantum secret sharing and quantum key distribution. Note that OTUH-QDS requires that all three participants have the bit correlations and before Alice signs the message, otherwise Bob and Charlie cannot successfully verify the signature. In our experiment, we use four-intensity decoy-state BB84 QKD to implement this bit correlation. Alice shares the secret keys and with Bob, and and with Charlie via QKD. Then, Alice gets her own secret keys by XOR operation. Suppose that Alice signs a $q$-bit document (message), denoted as $m$, and sends it to `forwarder' Bob.

*Messaging stage---*

1. *Signing--* Alice generates an irreducible polynomial of degree at random using a local quantum random number, which can be characterized by an -bit string . Then she uses her key bit string and the irreducible polynomial to generate a random linear feedback shift register-based (LFSR-based) Toeplitz matrix of rows and columns. She acquires a -bit digest . Here, is the digest of the -bit document through a hash operation with = , and is an -bit string for generating the irreducible polynomial in the LFSR-based Toeplitz matrix. Then, Alice encrypts the digest with her key bit string to obtain the -bit signature . She sends the document and signature . to Bob.
2. *Forwarding--* Bob transmits as well as his key bit strings to Charlie to inform Charlie that he has received the signature. Then, Charlie forwards his key bit strings to Bob. Bob obtains two new key bit strings by the XOR operation.
3. *Verification--* Bob exploits to obtain an expected digest and a string via XOR decryption. He utilizes and to establish an LFSR-based Toeplitz matrix and acquires an actual digest via a hash operation. Bob will accept the signature if the actual digest is equal to the expected digest. Then, he informs Charlie of the result. If Bob announces that he accepts the signature, Charlie creates two new key bit strings using his original key and the key sent by Bob. He employs to acquire an expected digest and a variable via XOR decryption. Charlie obtains an actual digest via a hash operation, where the hash function is an LFSR-based Toeplitz matrix generated by and . Charlie accepts the signature if the two digests are identical.

It needs to be mentioned that QDS requires only the quantum part of QKD protocols, also referred as key generation protocol (KGP). OTUH-QDS without perfect keys is different from the simple application of QKD, because the quantum keys of OTUGH-QDS do not need to perform privacy amplification that is one of the important steps of QKD, which will save computational resources, decrease delays, and decreasing failure probability.

*---Security of OTUH-QDS.*

This QDS protocol is naturally immune to repudiation and the probability of a successful forgery can be determined by

$$\varepsilon_{\mathrm{for}}=\left| m \right|{/2}^{p-1},$$

where $|m|$ is the length of the message. In this work, we choose $p=128$ and thus even for the -bit document it is still safe enough.}

1. **OTUH-QDS without perfect keys with BB84-KGP**

Recently, a variant of OTUH-QDS, called OTUH-QDS without perfect keys, was proposed~\cite{li2023one}. Different from OTUH-QDS that calls for sharing perfect quantum keys in the distribution stage, this variant uses share keys through KGP which is consist with that in single-bit QDS. In the following we introduce OTUH-QDS without perfect keys with BB84-KGP that is used in our experiment demonstration.

*Distribution stage---*

1. Alice-Bob and Alice-Charlie independently implement BB84-KGP to share correlated bit strings. This KGP process is the same as that in BB84-QDS. Thereafter, Alice-Bob and Alice-Charlie perform error correction algorithms on their shared bit strings. After this step, Alice holds two strings, denoted as and . She obtains one string through XOR operation . Bob and Charlie each holds one strings, denoted as and , respectively.
2. Alice randomly disturbs the orders of , and cuts the new string into -bit subgroups. The size of is estimated by parameters of BB84-KGP so that the security is guaranteed. Alice will publicize the new order and , and Bob and Charlie will perform the same operation on and accordingly.

*Messaging stage---*

The messaging stage is analogous to that in OTUH-QDS. One subgroup in the distribution stage contributes with length $P$ and another two subgroups contribute with length . The rules of Alice, Bob and Charlie are then consistent with that in OTUH-QDS.

*---Security of OTUH-QDS without perfect keys.*

This protocol is also naturally immune to repudiation attacks. The probability of a successful forgery is limited by

$$\varepsilon_{\mathrm{for}}=\left| m \right|\cdot2^{\left\{ 1-H_{P} \right\}},$$

where is the unknown information of a -bit subgroup generated in distribution stage, and can be estimated by parameters of BB84-KGP.

1. **Colluding attack**

Colluding attacks are the most serious problem in decentralized quantum digital signatures involving multiple participants~\cite{Weng2021secure}. A colluding attack means that there are more than two malicious nodes colluding together to disturb the normal functioning of a system. In our Byzantine agreement, due to complete decentralization, colluding attacks appear as the number of malicious nodes increases. In a three-party QDS, if the sender and forwarder are dishonest, they can collude together to make another node believe the forged message and the corresponding signature. In the broadcasting phase of our QBA protocol, this will lead to inconsistency of the delivered messages of the two adjacent multicast rounds without prejudice to the rule of coherence, as we can see in **Lemma 1** and **Lemma 2**. It allows dishonest players to deliver inconsistent messages in the system only under colluding attacks.

1. **Security analysis**

In our QBA protocol, the performance of honest (dishonest) players follows the same rule. Therefore, the players can be divided into two groups, the honest and dishonest. Also, the elements of a gathering list can be divided in the same way. Therefore, we can simplify the protocol with a perfect binary tree model where one tree node represents the set of multicast rounds with honest or dishonest primaries. The left (right) child tree node represents the multicast rounds with honest (dishonest) primaries of the next depth. In what follows, when we say a tree node is honest(dishonest), it means that the primaries in this tree node is honest (dishonest). And we can obtain the important **Lemma 1** and **Lemma 2**.

**Lemma 1:** *Suppose that B is a right child tree node of a parent node A who is honest, and C is the left child tree node of B. The messages delivered in C are consistent with those of A, which protects the consistency of the delivered messages.*

**Proof 1:** As shown in **Figure S1** (A), each primary of the honest node A multicasts to the backups. In this case, the dishonest forwarders cannot forward any messages except due to the unforgeability of the QDS, and each verifier receives .

Then, the coherence check guarantees that each primary of dishonest B must deliver to the honest forwarders in . Therefore, the messages multicast by the honest primaries of C in are , where marks the route before and containing A.

**Lemma 2:** *Suppose that B is a right child tree node of a parent node A who is honest, and E is the right child tree node of B. The message multicast in E can be inconsistent with those of A, which disrupts the consistency of the delivered messages.*

**Proof 2:** As shown in **Figure S1** (B), each primary of the honest node A multicasts to backups. Then, each primary of the dishonest node B can only deliver to the honest backups. However, the primaries of the dishonest B can execute the colluding attack together with the dishonest forwarders, and they can deliver any conflicting messages to the verifiers. After forwarding the different messages, these dishonest forwarders in ( marks the route before and containing A), who are also the dishonest primaries of E, can multicast these conflicting messages without compromising consistency. The consistency of the delivered messages is completely disrupted.


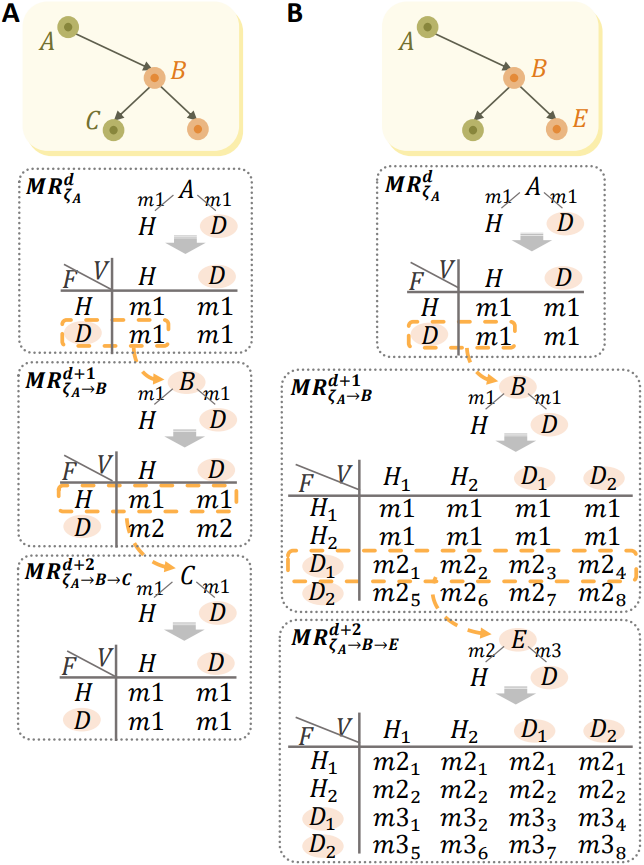


**Figure S1. Illustration of Lemma 1 and Lemma 2.** We use `F' to represent `forwarder', `V' to represent `verifier', `H' to represent `honest backups' and `D' to represent `dishonest backups' in the tables. The honest (dishonest) tree nodes are denoted by green (orange) nodes. The detailed process of the broadcasting phase is shown in tables where all recorded messages are summarized according to the honest and the dishonest case. **(A)** Illustration for Lemma 1. **(B)** Illustration for Lemma 2.

By Lemma 1 and Lemma 2, we find that on the route that avoids consecutively choosing the right child, the consistency of the delivered messages can be protected in the broadcasting phase. With this idea, we denote a special route as a safe path in the binary tree as follows.

**Definition 1:** The safe tree node, denoted by P, is defined as the first honest tree node in the message delivery route from the top to the bottom layer, as shown in **Figure S2** (A). Note that in the safe tree node, at least half of the backups are honest. Then, we continuously choose the left child node layer-by-layer until we reach an intermediate tree node. The intermediate node is defined as the tree node that has an equivalent number of honest and dishonest backups of depth , denoted by Q. As shown in **Figure S2** (C), from the intermediate tree node Q (depth ), we choose the right child K of Q (), the left child J of K (), the right child T of J (), the right child of T (), and so on. That is, the right and left child tree nodes are chosen in turn layer-by-layer until the ending tree node O of the penultimate depth is reached. This path from the safe tree node P, passing through the intermediate tree node Q, to reach the ending tree node O is defined as the safe path.


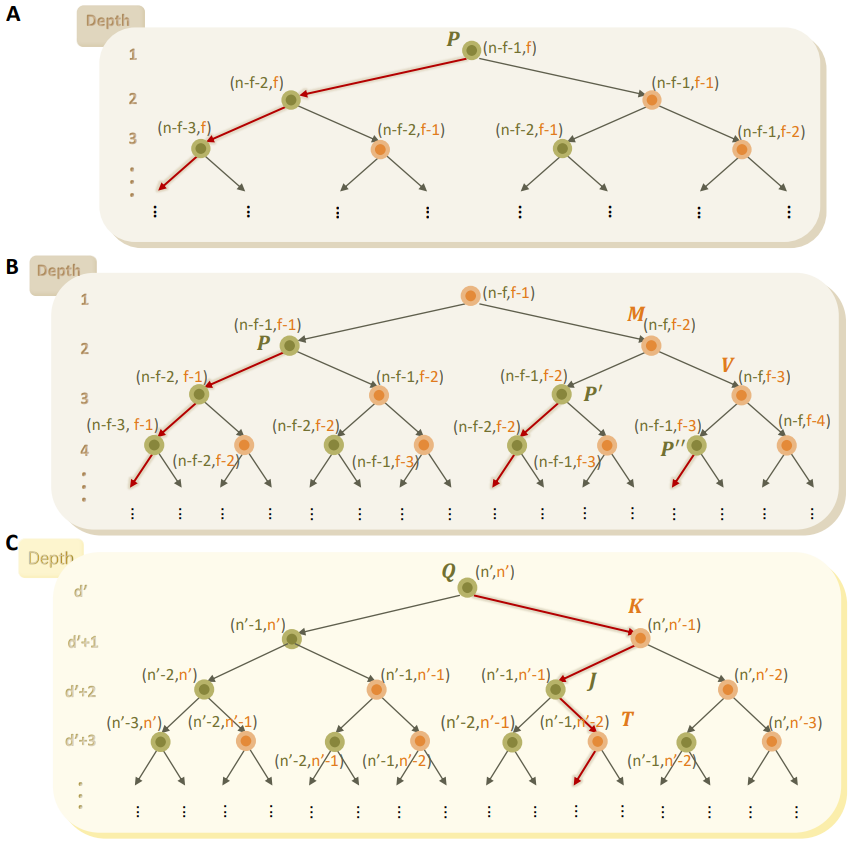


**Figure S2. The perfect binary tree model of our protocol and the safe path.** The green (orange) nodes in the tree represent the multicast rounds with honest (dishonest) primaries. Beside each node, the left number indicates the number of honest backups of this tree node, and the right number indicates the number of dishonest backups. The safe paths are represented by the red arrows. (A) and (B) illustrate the safe path before reaching the intermediate tree node. (A) The case in which the initial primary is honest. By the definition of a safe path, the honest initial primary is a safe node, denoted by P, and there is only one safe path in the whole process. (B) The case where the initial primary is dishonest and there is more than one safe path. The safe tree nodes are denoted by P, P, P and so on. (C) Illustration of the part of the safe path from the intermediate tree node Q to the ending tree node. The other tree nodes in the safe path are denoted by K, J, T and so on.

**Lemma 3:** *The safe path ensures that the honest players in the safe tree node can reach consensus on their outputs.*

**Proof 3:** In the broadcasting phase, by Lemma 1, the consistency of the message from the safe node's primaries can be protected. Considering one of the rounds of the safe tree node, the honest primary multicasts the message . For simplicity, the following discussion only analyses the route starting with this round. The analysis for the other rounds of the safe node is similar. On the safe path, the honest players in the subsequent rounds will receive and then multicast the message .

In the gathering phase, the message deducing process analyzed below demonstrates the consistency of the final outputs. From the safe tree node P to the intermediate tree node Q, more than half of the elements from the left (honest) child node appear in the gathering lists of each tree node. From the intermediate tree node Q to the ending tree node, if a tree node is honest, then in each gathering list of this node, the number of elements from the left child node is the same as that of the right child; if a tree node is dishonest, then in each gathering list of this node, the honest child node contributes one more element than the dishonest child node. Note that the output of each tree node in the safe path is determined by the tree nodes that are also on the safe path. Thus, all other branches in the binary tree can be ignored.

Our aim is to prove that in each tree node from the ending tree node to the safe tree node, more than half of the elements of each gathering list are always consistent, and thus the outputs of each node are always consistent during the recursion gathering process. We consider the two situations in **Figure S3**: (A) the ending tree node O is honest, and (B) the ending tree node O is dishonest. We denote the left (right) child tree node of O as . ().

**(a). The ending tree node O is honest.** We first analyze the outputs from the initial gathering lists of and . The message of is , which is also the message that is multicast in the safe node. In the bottom layer, each backup has the same gathering list where all the elements are , which is obtained directly from the bottom broadcasting list. Thus, the honest backups of have the same output. The primaries of multicast to the honest backups. Since the honest backups contribute one element more than the dishonest backups in each gathering list, message is the majority. Thus, each honest backup of has the same output . That is, the outputs from and are all . Then, the honest backups of the ending tree node O have the same output deduced from their consistent gathering lists.

Considering the consistent outputs of the ending tree node, consistency can always be held on the safe path. Suppose that node U is the dishonest parent tree node of O at . The output of each gathering list of U is determined by elements from O. Thus, the outputs are also . On the safe path, the parent tree node of U has at least half of the elements, which are , in each gathering list. Moreover, for a certain backup's gathering list, there is also one element from the backup himself or herself, which is the message he or she received directly from the corresponding primary. This element is also by Lemma 1. Therefore, more than half of the elements in each gathering list are , and the output is . Following the above process until the intermediate tree node Q is reached, we can see that all the outputs of the tree nodes on the safe path are . Finally, the honest backups of node Q have the same output .

In the tree nodes from P to Q, for each gathering list, more than half of the elements are . Finally, in the safe tree node P, all honest players in each round will reach consensus.

**(b). The ending tree node O is dishonest.** The output of O is determined by the elements from . We find that these elements are all since is honest. Thus, the backups in the ending tree node O have the same output . Similar to the analysis in (a), each backup of the intermediate node Q outputs . Therefore, all the honest players in the safe tree node P have consistent outputs .


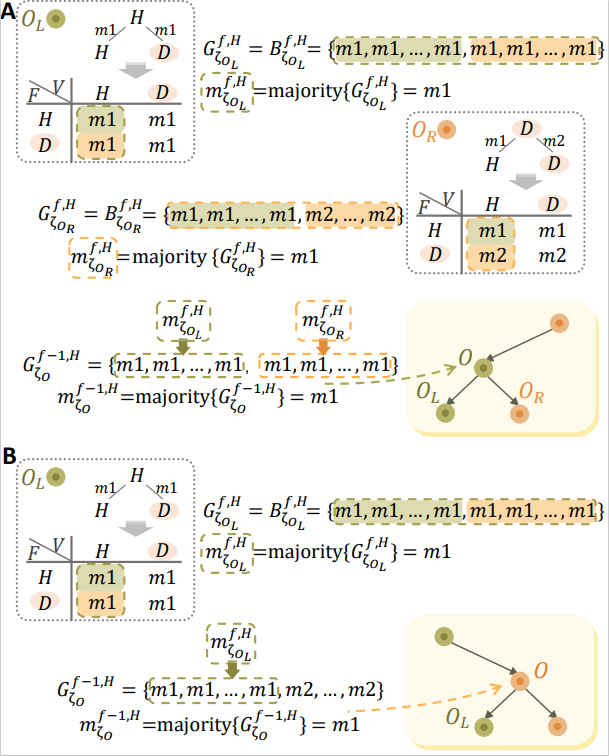


**Figure S3. The ending tree node of a safe path at the penultimate depth and its child tree nodes.** The yellow figure of the binary tree presents the ending tree node of a safe path, denoted by O, and its left (right) child tree node is denoted by (). The messages contributed by the honest (dishonest) backups are marked by green (orange). The messages recorded in the broadcasting list are represented in the table. The gathering list and the corresponding outputs are also presented beside the tables. **(A)** The ending tree node is honest. **(B)** The ending tree node is dishonest.

**Theorem 1:** For an $n$-player system with $f$ malicious players, our QBA protocol can reach consensus with a fault tolerance of $N\geq2f+1$.

**Proof:** We start with . We analyze it according to whether the initial primary is honest or dishonest.

**(a) The initial primary is honest.** By Definition 1, the root tree node is not only the safe tree node but also the immediate tree node. By Lemma 3, all honest players in the initial round can reach consensus, which satisfies the Byzantine conditions $IC_{1}$ and $IC_{2}$.

**(b). The initial primary is dishonest.** As shown in **Figure S2** (B), the dishonest initial primary S can arbitrarily deliver different messages to different forwarders at depth 1. At depth 2, the left (honest) child tree node is a safe tree node that starts a safe path. The primaries of the right (dishonest) child tree node M can execute colluding attacks and deliver conflicting messages as described in Lemma 2. The left child tree node P of M is another safe tree node that starts another safe path. Similarly, the tree node P is also a safe tree node that starts another safe path, and so on.

One of the honest player's outputs in the initial round is , where . By Proof 3, although the outputs of the tree node P may be different, the honest players reach consensus on each of these outputs since P is a safe tree node. Thus, in the gathering list , the elements from the safe tree node P are consistent among the honest backups. Next, we discuss the elements of the list from node M, as shown in **Figure S2** (B). Similar to the above process, we can find that all honest backups reach consensus on the elements from the safe tree node P, so we must consider the elements of the list from node V, and so on. After we continuously choose the right child tree node at the next depth, the dishonest backups of the tree nodes will continuously be reduced by one while the number of honest backups will not change. When we reach depth , in the dishonest leaf node, only the primary is dishonest and all the backups are honest. Thus, there are no colluding attacks. Although the outputs of different rounds may be different, the backups of the same round can obtain consistent outputs.

In the dishonest parent of this leaf node, each gathering list has one element from this dishonest leaf node (right child node) and elements from the honest leaf node (left child node).

The left child node is also a safe node and these elements are also consistent. Thus, each honest backup of this dishonest parent tree node also obtains a consistent output. Following the above recursion process, we find that the honest backups in each round of the above path always have the same gathering lists. The consistent outputs from each safe node indirectly or directly lead to the eventual consistency of the elements that make up each gathering list of .

In the initial round, the elements in a list may be different from each other, but the gathering lists of the honest players are the same, regardless of the messages delivered by the dishonest primary. Finally, all the honest players reach consensus and output consistent messages, which satisfies the condition $IC_{2}$.

In summary, we prove that our protocol can satisfy the two Byzantine conditions, $IC_{1}$ and $IC_{2}$, to reach Byzantine agreement when .

If , then the safe paths appear too late in the binary tree model. The consistency of the delivered messages cannot be guaranteed in the tree nodes before the safe path. For example, when and the initial primary is honest. The root tree node is honest, but the number of honest backups is and the number of dishonest backups is . Therefore, the root tree node is no longer a safe tree node. In fact, the minimum depth at which we can find a safe tree node is in the binary tree. There are two safe nodes, denoted as and , that begin their safe paths at . and can be found by the following steps. : After choosing the right child tree node twice, the left tree node at depth 4 is . : First choosing the left child of the root tree node first, and then choosing the right tree node, finally the left tree node at depth 4 is . By Lemma 2, the message multicast in can conflict with the message delivered by the initial primary. Suppose the messages multicast by the initial primary are and the conflicting messages delivered by dishonest players are . Then the backups of tree node will consistently output . The backups of the tree node will still consistently output . After several rounds of counting, the numbers of messages and in his or her own gathering list for the initial round are and , respectively. Then, all the honest backups in the initial round will output , while the honest initial primary outputs . Therefore, they cannot reach consensus. When , the situation will undoubtedly worsen.

1. **Experiment calculation details**

We experimentally implement the three-party consensus utilizing GC01-QDS, OTUH-QDS, and OTUH-QDS without perfect keys, respectively, and implement the five-party consensus with OTUH-QDS. Here, we utilize four-intensity decoy-state BB84 key generation process for the three QDS protocols. The correlated quantum keys of different pairwise users are pre-distributed in the laboratory via a fibre spool. The five users bring their own secret keys. We choose player , and to perform three-party consensus and choose all five nodes to perform five-party consensus. The decentralized digital ledger is shown in Fig.~\ref{receipt}, which is reached consensus on by the users in the experiment. The digital ledger is converted into a binary string of bits. We denote this correct message as , and the incorrect messages as , , and so on.


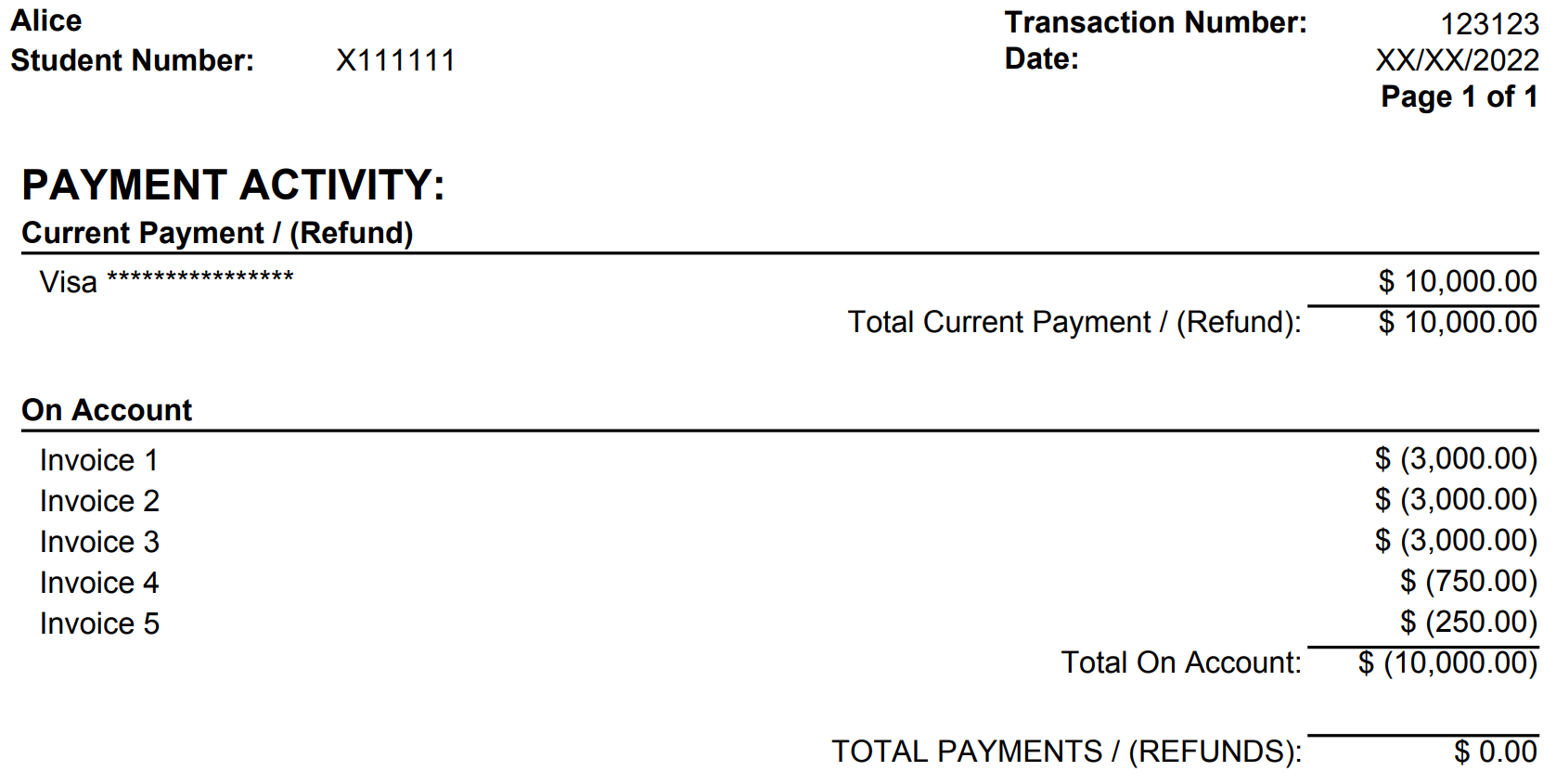


**Figure S4 The digital ledger for transmission in the experiment.** We convert the digital ledger into a binary string of bits. The binary string of bits is the actual message we transmit in the experiment.

We will introduce the calculation details based on our experiment.

() denotes the upper (lower) bound of the observed value $x$.

Using the decoy-state method for finite sample sizes, the expected number of vacuum events and single-photon events . can be expressed as

and

respectively. Here is the count of () intensity pulse measured in the Z(X) basis, and is the expected value of observed value . We use the variant of the Chernoff bound~\cite{yin2020tight} to obtain the lower and upper bounds, and , where .

The expected value of the number of single-photon events in can be given by

Additionally, the expected number of bit errors associated with the single-photon event in is , where . For a given expected value , the upper and lower bounds of the observed value are given by and , respectively. Using random sampling without replacement, the phase error rate in the Z basis is

where , and .

1. **Single-bit GC01-QDS**

In BB84-KGP GC01-QDS, the unknown information to the attacker is given by

where .

According to the , we can obtain the signature rate

where MHz is the effective repetition rate, is the length of the message, is the minimum number of pulses required to securely sign a one-bit message according to the set security parameter.

1. **OTUH-QDS**

The length of the final key, which is -correct and -secret, can be expressed by

and the signature rate of BB84-KGP OTUH-QDS can be expressed as

where and is the time of sending pulses using 96 MHZ repetition rate.

1. **OTUH-QDS without perfect keys**

In OTUH-QDS without perfect keys based on BB84-KGP, Alice and Bob (Alice and Charlie) form the -length raw key bit from the random bits under the Z basis. We can estimate parameters in a selected P-bit group, i.e., the lower bound of number of vacuum events and single-photon events under the Z basis and , and the upper bound of the phase error rate of the single-photon events in the Z basis .

Finally, we can obtain the unknown information of the P-bit group

According to the and the set security parameter, we can obtain the signature rate

where is the minimum number with the condition

satisfied, where is upper bound of the failure probability of the QDS protocol.
